# Vascular progenitor cell senescence in patients with Marfan syndrome

**DOI:** 10.1111/jcmm.14301

**Published:** 2019-03-28

**Authors:** Haiwei He, Baoqi Yu, Zipeng Liu, Gen Ye, Wei You, Yimei Hong, Qizhou Lian, Yuelin Zhang, Xin Li

**Affiliations:** ^1^ School of Medicine South China University of Technology Guangzhou China; ^2^ Department of Emergency Medicine Department of Emergency and Critical Care Medicine Guangdong Provincial People's Hospital Guangdong Academy of Medical Sciences Guangzhou China; ^3^ Department of Physiology and Pathophysiology School of Basic Medical Sciences Capital Medical University Key Laboratory of Remodelling‐Related Cardiovascular Diseases Ministry of Education Beijing China; ^4^ Center for Genomic Sciences LKS Faculty of Medicine the University of Hong Kong Hong Kong SAR China; ^5^ Department of Medicine LKS Faculty of Medicine the University of Hong Kong Hong Kong SAR China

**Keywords:** Marfan syndrome, mitochondria dynamics, senescence, transforming growth factor β, vascular progenitor cells

## Abstract

Vascular progenitor cells (VPCs) present in the adventitia of the vessel wall play a critical role in the regulation of vascular repair following injury. This study aimed to assess the function of VPCs isolated from patients with Marfan syndrome (MFS). VPCs were isolated from control and MFS donors and characterized. Compared with control‐VPCs, MFS‐VPCs exhibited cellular senescence as demonstrated by increased cell size, higher SA‐β‐gal activity and elevated levels of p53 and p21. RNA sequencing showed that several cellular process‐related pathways including cell cycle and cellular senescence were significantly enriched in MFP‐VPCs. Notably, the expression level of TGF‐β1 was much higher in MFS‐VPCs than control‐VPCs. Treatment of control‐VPCs with TGF‐β1 significantly enhanced mitochondrial reactive oxidative species (ROS) and induced cellular senescence whereas inhibition of ROS reversed these effects. MFS‐VPCs displayed increased mitochondrial fusion and decreased mitochondrial fission. Treatment of control‐VPCs with TGF‐β1 increased mitochondrial fusion and reduced mitochondrial fission. Nonetheless, treatment of mitofusin2 (Mfn2)‐siRNA inhibited TGF‐β1‐induced mitochondrial fusion and cellular senescence. Furthermore, TGF‐β1‐induced mitochondrial fusion was mediated by the AMPK signalling pathway. Our study shows that TGF‐β1 induces VPC senescence in patients with MFS by mediating mitochondrial dynamics via the AMPK signalling pathway.

## 1 INTRODUCTION

Marfan syndrome (MFS) is mainly caused by the fibrillin‐1 (FBN1) gene mutation and is a hereditary disorder of connective tissue with effects on multiple systems including cardiovascular, skeletal and ocular.^1^ FBN1 encodes the extracellular matrix protein FBN1 and is involved in the formation of complex extracellular structures in the arteries. FBN1 mutation can lead to contractile dysfunction of smooth muscle cells (SMCs) with consequent reduced tensile strength of aortic tissue.^2^ Aortic complications such as aortic dilatation and dissection are the main cause of morbidity and mortality in patients with MFS.^3^ Although considerable progress has been made over the past decades in the treatment of MFS‐induced cardiovascular injury, including medical and surgical interventions, efficacy has been limited due to the unclear molecular aetiologies. An understanding of the fundamental molecular mechanisms that underlie MFS will provide a novel strategy for MFS management.

The wall of the aorta is composed of three layers: intima, media and adventitia. Recent research has revealed a range of vascular progenitor cells (VPCs) in the adventitia of the vessel wall that are positive for stem cell antigen‐1(Sca‐1) or c‐kit.^4^, ^5^, ^6^ These VPCs can give rise to many cell lineages, including endothelial cells and SMCs.^7^, ^8^ It has been well documented that VPCs contribute to cardiovascular regeneration therapies.^5^ Transplantation of VPCs isolated from the adventitia of patients with coronary artery bypass graft has been shown to greatly enhance blood perfusion recovery and neovascularization in a mouse model of hindlimb ischaemia,^9^ demonstrating their great potential in vascular disease treatment. Nevertheless, the function of stem cells usually declines in a disease milieu.^10^ It remains unclear whether the function of VPCs declines in patients with MFS.

A FBN1 defect activates transforming growth factor beta (TGF‐β) signalling, leading to vascular injury.^11^, ^12^, ^13^ Accumulating evidence has shown that TGF‐β is involved in regulation of stem cell senescence. Treatment with TGF‐β can induce cellular senescence of young MSCs but treatment with anti‐TGF‐β antibodies can reduce cellular senescence of old MSCs.^14^ Inhibition of TGF‐β1 signalling significantly inhibits serum‐free‐induced endothelial cell senescence and thereby improves endothelial function.^15^ Disrupted mitochondrial dynamics that are regulated by fusion and fission are strongly associated with cellular senescence.^16^, ^17^ Mitochondrial fission is mainly regulated by dynamin‐related protein 1 (Drp1) and mitochondrial fission factor (Mff) whereas mitochondrial fusion is mediated by mitofusion (Mfn) 1, Mfn2 and optic atrophy 1 (Opa1) proteins. Whether TGF‐β1 induces VPC senescence via regulation of mitochondrial dynamics and the underlying mechanisms have not been determined. In this study, we revealed that VPCs isolated from MFS exhibited cellular senescence. Importantly, we found that TGF‐β1 disrupted mitochondrial dynamics via regulation of the adenosine monophosphate‐activated protein kinase (AMPK) signalling pathway, leading to VPC senescence in MFS patients.

## 2 MATERIALS AND METHODS

### 2.1 C‐kit VPC isolation, culture and characterization

Vascular progenitor cells were isolated from control donors and patients with MFS at Guangdong Provincial People's Hospital, China. Written informed consent was obtained from all study patients and detailed information is summarized in Table 1. This study was approved by the research ethics board of Guangdong Provincial People's Hospital.

**Table 1:** Demographic characteristics of the study patients

	Control	MFS	*P*‐value
Total patients	11	10	—
Age (y), mean ± SEM	35.00 ± 4.072	30.40 ± 2.613	0.3644
Male, n (%)	9 (81.8%)	8 (80.0%)	—
Height (cm), mean ± SEM	165.2 ± 3.468	178.8 ± 2.272	0.0081
Weight (kg), mean ± SEM	58.33 ± 4.828	63.33 ± 4.008	0.4440
BMI (kg/m^2^), mean ± SEM	21.20 ± 1.205	19.87 ± 1.427	0.4950
BSA (m^2^), mean ± SEM	1.601 ± 0.081	1.749 ± 0.051	0.1540

BMI, body mass index; BSA, body surface area; MFS, Marfan syndrome.

BSA = 0.0061 * height (cm) + 0.0128 * weight (kg) − 0.1529.

Human VPCs were isolated, sorted and cultured as previously described.^18^ Briefly, aorta specimens were taken from the ascending aorta of control donors and MFS patients. After removing the adipose tissue, the adventitial tissue was separated from the media and intima. The adventitia was cut into 1‐2 mm^3^ pieces and digested in Liberase TL (Roche, 5401020001) at 37°C for 3.5 hours, then passed through a 100 μm and 70 μm cell strainer to obtain cell suspension. VPCs were sorted using anti‐C‐kit immunomagnetic microbeads (Miltenyi Biotec, 130‐091‐332) and a magnetic cell separator (Miltenyi Biotec, 130‐042‐201), then cultured in DMEM/F12 containing 15% FBS, streptomycin and penicillin 100 U/mL and leukaemia inhibitory factor (LIF) 10 ng/ml. The VPCs with passage 3‐4 were used in the current study. Both control‐VPCs and MFS‐VPCs were passaged at 3‐day intervals and the same cell number (100 000 cells per 6‐cm dish) was plated. Population doubling was measured at each passage.

The surface antigen expression of VPCs was examined by flow cytometry. The antibodies including anti‐c‐kit (Biolegend, 313205), anti‐CD29 (Biolegend, 303003), anti‐CD73 (Biolegend, 344003), anti‐CD105 (Biolegend, 323205), anti‐CD90 (Biolegend, 328107), anti‐CD31 (Biolegend, 303111) and anti‐CD45 (Biolegend, 304011) were used.

The differentiation capacity of VPCs into SMCs, osteocytes and adipocytes was evaluated as previously described.^19^, ^20^

### 2.2 Scratch‐wound assay

Control‐VPCs and MFS‐VPCs were seeded in a 12‐well plate and cultured with complete culture media. When cells reached 90% confluence, scratches of the same width were made on the bottom of the plate using a 1 mL pipette tip. Cells were carefully washed with PBS to remove cell debris and then incubated with serum‐free medium in an incubator with 5% CO_2_ at 37°C. After 24 hours incubation, the migration of VPCs into the ‘wound’ area was evaluated by a phase contrast microscope.

### 2.3 Transwell assay

The migration capacity of VPCs from different groups was assessed by transwell inserts with 8.0 μm micron pore membrane filters in a 24‐well plate (#3422, Corning Life Science). Briefly, VPCs from different groups (3 × 10^4^ cells/well) were seeded on the upper chamber of Transwell with the VPC medium containing 2% FBS. Meanwhile, the VPC medium containing 5% FBS was added to the lower chamber. After 24‐hour incubation at 37°C with 5% CO_2_, migratory VPCs were fixed with formaldehyde for 15 minutes and then stained followed by addition of 1% crystal violet at room temperature for 15 minutes. Data are representative of mean cell number of migratory VPCs in six random fields at 20× magnification.

### 2.4 Senescence‐associated β‐galactosidase (SA‐β‐gal) staining

The cellular senescence of VPCs was evaluated by SA‐β‐gal staining according to the manufacturer's protocol (C0602, Beyotime). Briefly, VPCs were seeded in a 6‐well plate and given different treatments. After washing with PBS three times, VPCs were fixed with fixative solution for 15 minutes and then incubated overnight with SA‐β‐gal staining solution at 37°C (without CO_2_). The percentage of senescent VPCs stained blue was assessed from five different view fields of each sample in three independent experiments.

### 2.5 Measurement of mitochondrial reactive oxygen species

Mitochondrial reactive oxygen species (ROS) in VPCs were examined by Mito‐Sox staining (Invitrogen, M36008). Briefly, VPCs were seeded in 24‐well plates with glass coverslips. After different treatments, VPCs were washed with PBS and incubated with 5 μmol/L Mito‐sox at 37 °C for 15 minutes in the dark. Subsequently, the VPCs from different treatments were randomly photographed. Images of five different view fields for each slide were captured randomly by a motorized inverted microscope (Olympus, Hamburg, Germany) at 535‐nm excitation and 610‐nm emission wavelengths. The fluorescence intensity was analysed using Image J software (National Institutes of Health, Bethesda, MD, USA) in three independent experiments. The level of mitochondrial ROS was normalized by the fluorescence intensity of control cells.

### 2.6 Western blotting

The proteins of VPCs with different treatments were extracted using RIPA (CST, 9806) with Protease/Phosphatase inhibitor (CST, 5872) and the concentrations determined using the bicinchoninic acid (BCA) assay kit (Thermo, 231227). A total of 30 μg protein lysate from each sample was loaded, separated by SDS/PAGE and then transferred to a PVDF membrane. After blocking with 5% fat free milk in TBST, the PVDF membrane was incubated overnight at 4°C with the following antibodies: anti‐TGF‐β1 (Abcam, ab64715), anti‐p53 (Abcam, ab26), anti‐p21 (Abcam, ab109199), anti‐p‐Drp1 ser616 (Invitrogen, PA5‐64821), anti‐Drp1 (Invitrogen, PA5‐20176), anti‐Mfn2 (Abcam, ab124773), anti‐p‐AMPK (CST, 4184), anti‐AMPK (CST, 5832), anti‐Smad2 (SC‐101153), p‐Smad2 (CST, 3108s) and GAPDH (CST, 2118). Next, the membrane was washed three times with TBST and incubated with secondary antibodies (1:3000, CST) at room temperature for 1 hour and then exposed in a dark room.

### 2.7 RNA sequencing and RNA‐seq data processing

RNA‐seq analysis was performed on control‐VPCs and MFS‐VPCs using the Illumina sequencing platform and RNA‐seq data processed as described previously.^21^, ^22^ In brief, differentially expressed genes (DEG) were defined by an absolute value of log1.5 (fold change) > 1 and adjusted *P*‐value <0.05. Pathway enrichment analysis was performed with significant DEGs from the sequencing results. Pathways with a *P*‐value <0.01 were considered significant results. For each pathway, we then calculated the ratio of the number of significant DEGs to the number of total sequenced genes involved in the corresponding pathway. We selected significant DEGs that were enriched in the cellular senescence pathway to plot the heatmap. Unknown genes and genes with missing expression in more than two samples were discarded.

### 2.8 MitoTracker staining

The mitochondrial morphology of VPCs was examined by MitoTracker Green FM (Invitrogen, M7514). Briefly, VPCs from different groups were incubated in the dark for 30 minutes with DMEM supplemented with 20 nmol/L MitoTracker Green FM. After washing with PBS three times, cells were mounted with 4′, 6‐diamidino‐2‐phenylindole (DAPI; Vector Laboratories, Inc.) and photographed using a confocal microscope.

### 2.9 Immunofluorescence staining

Immunofluorescence staining was carried out according to the protocol as previously described.^23^ Briefly, control‐VPCs and MFS‐VPCs were fixed with formaldehyde for half an hour. Following permeation with 0.1% Triton X‐100 in PBS for 30 minutes, cells were stained with ki‐67 antibody (Abcam, ab15580), c‐kit antibody (Abcam, ab32363), γH2AX antibody (Abcam, ab81299) and incubated overnight at 4°C with a 1:100 dilution. After washing with PBS three times, cells were incubated with the secondary antibodies. Finally, the sample was mounted with DAPI and photographed. Images of five different view fields for each slide were captured randomly by a motorized inverted microscope and analysed using AxioVision (Zeiss). The percentage of positive cells was calculated in three independent experiments.

### 2.10 Small‐interfering RNA (siRNA) silencing

TGF‐β1‐siRNA (sc‐44146), Mfn2‐siRNA(sc‐43928) and control siRNA(sc‐37007) were used to transfect VPCs using a Lipofectamine RNAiMAX Reagent Kit (Invitrogen, 13778030) according to the protocol. Seventy‐two hours after transfection, VPCs were collected and the silencing efficiency was examined by Western blotting.

### 2.11 Statistical analysis

All data are expressed as the mean ± SEM. Statistical analyses were performed with Prism 5.04 Software (GraphPad Software for Windows, San Diego, CA, USA). Comparison between two groups was analysed by unpaired Student's *t* test and between multiple groups by one‐way ANOVA followed by the Bonferroni test. A *P*‐value <0.05 was considered statistically significant.

## 3 RESULTS

### 3.1 Characterization of VPCs

**Figure 1:**
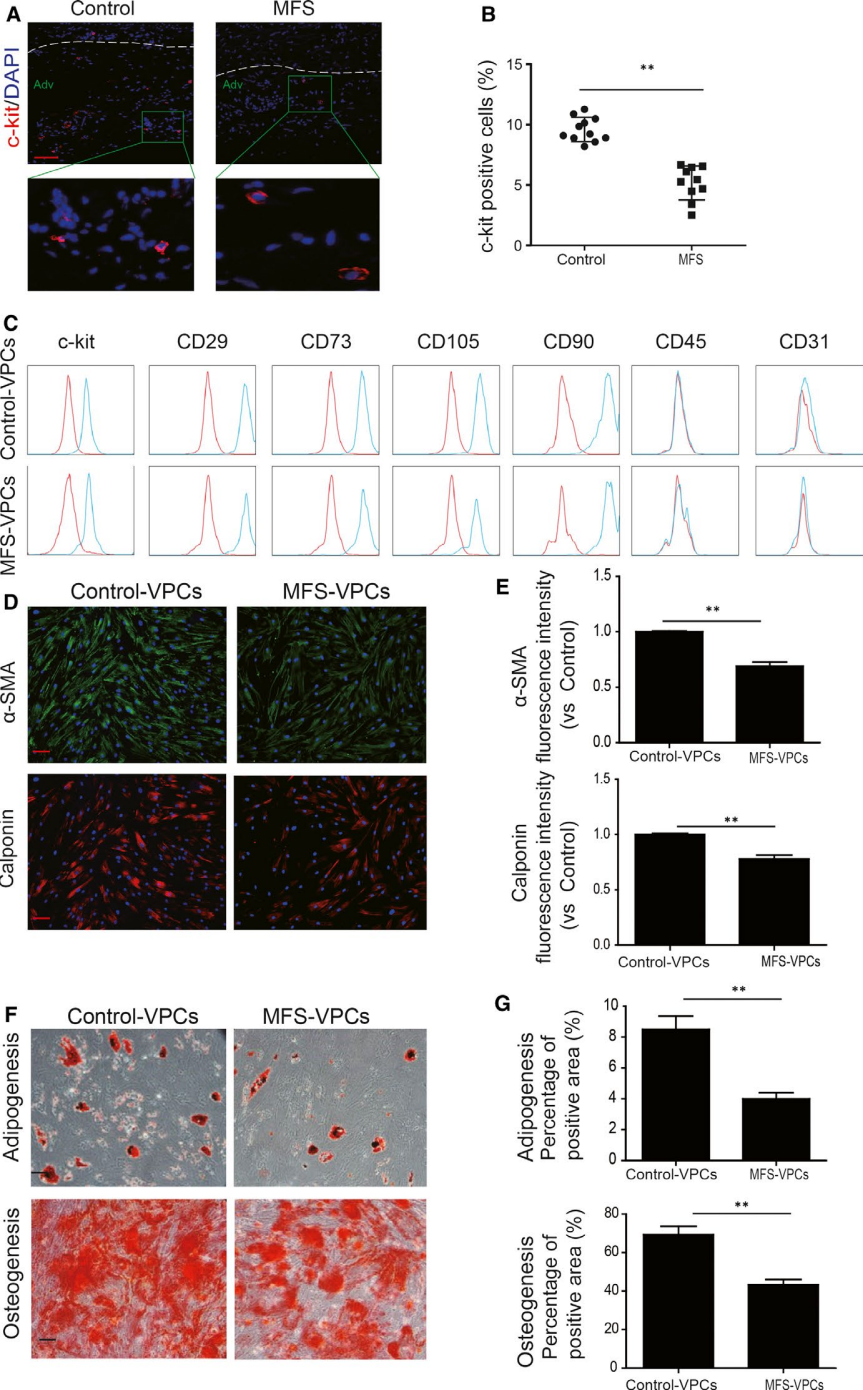
Characterization of vascular progenitor cells (VPCs). A, Representative images of c‐kit staining in the adventitia of aorta from control and Marfan syndrome (MFS) donors. B, The c‐kit positive cells in the adventitia of the aorta from control and MFS donors were calculated and are presented as a percentage of total cells. C, Surface marker profiling determined by fluorescence‐activated cell sorting (FACS) in VPCs. that is, positive for c‐kit, CD29, CD73, CD90, CD105; negative for CD31, CD45. D, Representative images of α‐SMA and Calponin staining showing VPCs differentiated into smooth muscle cells. F, The differentiation capacities of adipogenesis and osteogenesis of VPCs in vitro were determined by Oil red staining and Alizarin red staining respectively. E, Quantitative analysis of fluorescence intensity of α‐SMA and Calponin in control‐VPCs and MFS‐VPCs after differentiation. G, Quantification of the area occupied by the oil red O staining and Alizarin red staining in control‐VPCs and MFS‐VPCs after differentiation. Data are expressed as mean ± SEM. **P < 0.01. Scale bar = 100 μm

Previous studies have shown the existence of VPCs that express Sca‐1, CD34 and C‐kit in the adventitia of the vessel wall.^24^, ^25^ We examined VPCs in the aorta isolated from control donors and MFS donors by c‐kit immunofluorescent staining. The results showed that c‐kit positive cells were presented in the adventitia of aorta from both control donors and MFS donors (Figure 1A). Furthermore, compared with control donors, the number of c‐kit positive VPCs was greatly reduced in MFS patients (Figure 1B). Next, the c‐kit positive cells were isolated from the adventitia and cultured. The surface profile of VPCs was examined by flow cytometry. Both control‐VPCs and MFS‐VPCs had similar surface markers, that is, c‐kit (+), CD29 (+), CD73 (+), CD90 (+), CD105 (+), CD45 (−), CD31 (−) (Figure 1C). In addition, immunofluorescent staining showed that VPCs can differentiate into SMCs as demonstrated by α‐SMA and Calponin (Figure 1D). The expression levels of both α‐SMA and Calponin were significantly reduced in MFS‐VPCs compared with control‐VPCs (Figure 1E). Moreover, both control‐VPCs and MFS‐VPCs were able to differentiate into adipocytes and osteocytes in vitro (Figure 1F). Importantly, compared with control‐VPCs, the differentiated capacity of MFS‐VPCs into adipocytes and osteocytes was significantly reduced, indicating that the function of MFS‐VPCs was impaired (Figure 1G).

### 3.2 VPCs isolated from patients with MFS exhibit cellular senescence

**Figure 2:**
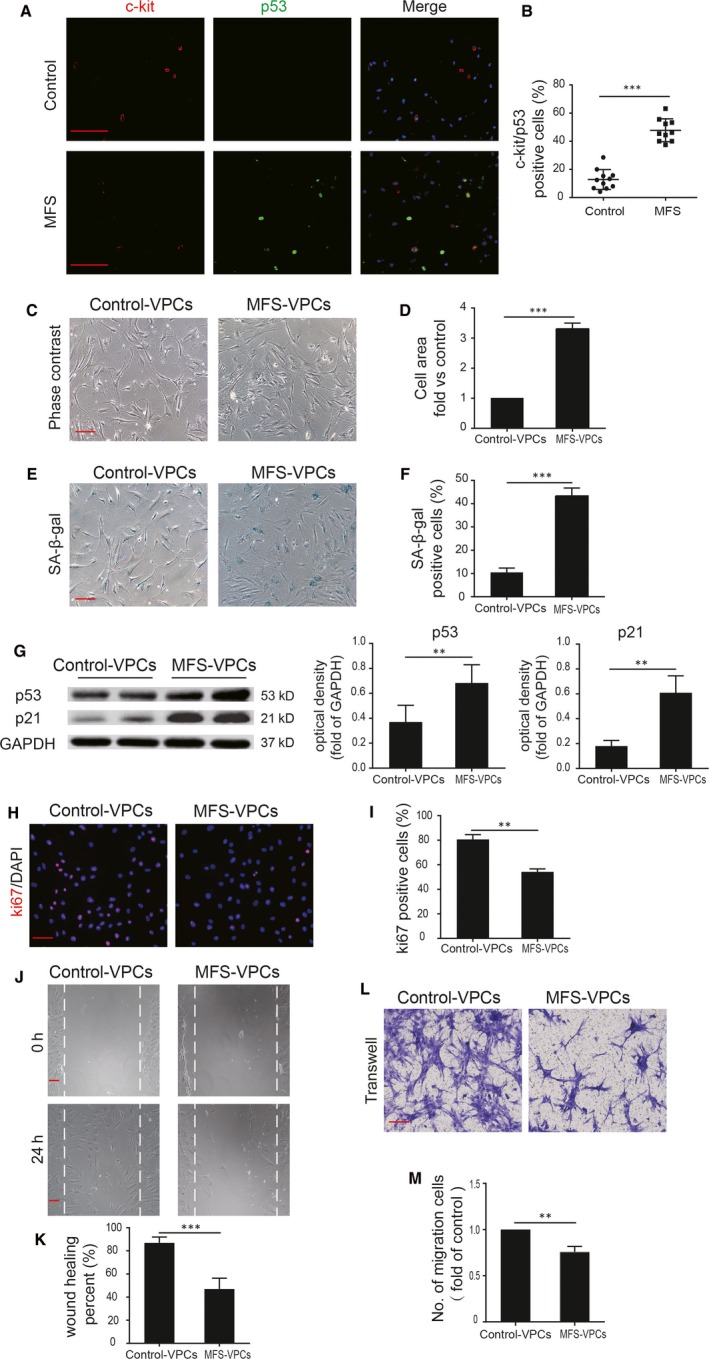
Marfan syndrome (MFS)‐vascular progenitor cells (VPCs) exhibit cellular senescence. A, Representative images of c‐kit and p53 staining in the ascending aorta of control donors and MFS patients. B, The c‐kit and p53 double positive cells in the ascending aorta of control donors and MFS patients were calculated and are presented as a percentage of total cells. C, Representative images of control‐VPCs and MFS‐VPCs under light microscopy. Images of five different view fields for each slide were captured. D, The cell size of control‐VPCs and MFS‐VPCs was calculated using Image J software. The cell size was measured relative to the control. E, Representative images of SA‐β‐gal staining in control‐VPCs and MFS‐VPCs. F, The SA‐β‐gal positive cells in control‐VPCs and MFS‐VPCs were calculated and are presented as a percentage of total cells. G, Western blotting and quantitative analysis of the level of p53 and p21 protein in control‐VPCs and MFS‐VPCs. H, Representative images of Ki‐67 staining in control‐VPCs and MFS‐VPCs. I, The Ki‐67 positive cells in control‐VPCs and MFS‐VPCs were calculated and are presented as a percentage of total cells. J, Representative images of wound healing assay showing the migratory capacity of control‐VPCs and MFS‐VPCs. K, The wound recovery rate of control‐VPCs and MFS‐VPCs was quantified. L, Representative images of transwell assay showing the invasive capacity of control‐VPCs and MFS‐VPCs. M, The invasive capacity of control‐VPCs and MFS‐VPCs was calculated. Data are expressed as mean ± SEM (n = 3). **P < 0.01, ***P < 0.001. Scale bar = 100 μm

Cellular senescence is closely associated with reduced cell function including reduced differentiation capacity and decreased proliferation.^26^ Therefore, we first conducted immunofluorescent assay to identify senescent VPCs in the ascending aorta of control donors and MFS patients. As shown in Figure 2A, some c‐kit positive cells were co‐stained with p53, a senescence‐associated marker, indicating the senescent VPCs (Figure 2A). Notably, more c‐kit and p53 double positive cells were observed in the ascending aorta of MFS patients compared with control donors (Figure 2B). Next, we isolated the c‐kit positive cells from the donors and cultured, and then immediately examined the cellular senescence of VPCs without expansion. We found that compared with control‐VPCs, the senescence of MFS‐VPCs was greatly enhanced as determined by SA‐β‐gal staining (Figure S1). Notably, the senescence of VPCs was not increased when expanded to passage 4, indicating that no obvious replicative senescence of VPCs occurs less than passage 4 (Figure S1). Moreover, the population doubling time of control‐VPCs and MFS‐VPCs were similar until passage 6. The population doubling time was greatly increased in MFS‐VPCs at passage 6 and in control‐VPCs at passage 15. To keep enough VPCs for the experiment, we used the VPCs at passage 3‐4. We examined the morphology of the two types of cells in vitro. The results showed that control‐VPCs exhibited a healthy spindle shape but MFS‐VPCs were enlarged and flattened (Figure 2C). MFS‐VPCs showed an increased cell size compared with control‐VPCs (Figure 2D). SA‐β‐gal analysis demonstrated that MFS‐VPCs exhibited more SA‐β‐gal positive cells (Figure 2E,F). The protein level of senescence‐associated markers (p53, p21) was also markedly increased in MFS‐VPCs compared with control‐VPCs (Figure 2G). Furthermore, ki‐67 staining showed that the number of ki‐67 positive cells was dramatically reduced in MFS‐VPCs compared with control‐VPCs, suggesting that the proliferative capacity of MFS‐VPCs was decreased (Figure 2H,I). We also performed wound healing and transwell assays to evaluate the migration ability of control‐VPCs and MFS‐VPCs (Figure 2J,L). The number of migrating and invading cells was significantly reduced in MFS‐VPCs (Figure 2L,M). Accumulation of DNA damage can lead to cell senescence, we therefore detected DNA damage in VPCs from MFS patients and control donors by determination of γH2AX. The results showed that compared with control donors, the percentage of γH2AX positive cells was greatly increased in VPCs from MFS patients (Figure S2A,B). These findings suggest that VPCs isolated from patients with MFS exhibit cellular senescence.

### 3.3 Transcriptomic comparison of control‐VPCs and MFS‐VPCs

**Figure 3:**
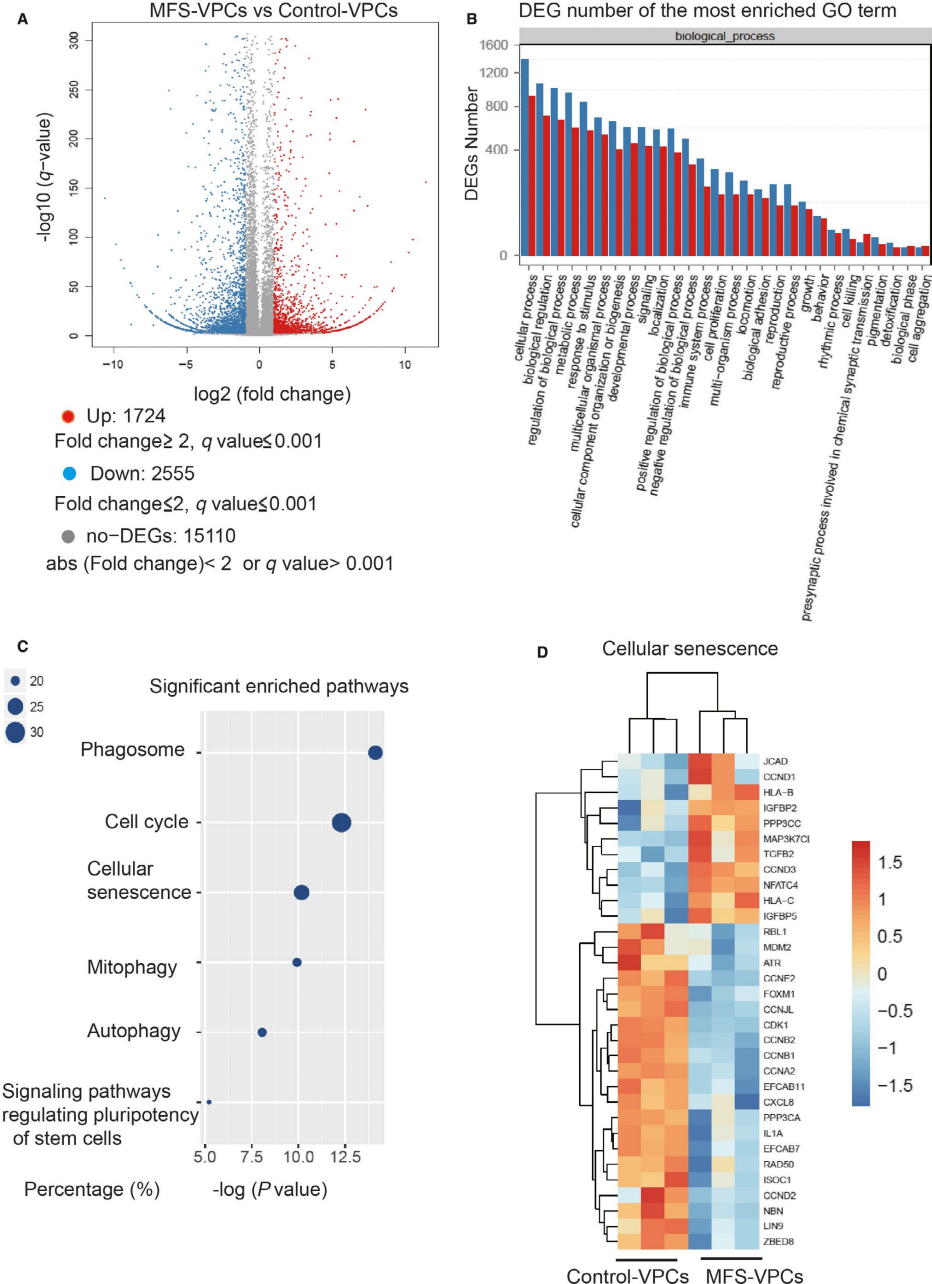
Gene expression profile analysis of control‐vascular progenitor cells (VPCs) and Marfan syndrome (MFS)‐VPCs. A, Volcano plot showing the number of up‐regulated and down‐regulated genes in MFS‐VPCs compared with control‐VPCs. B, Differentially expressed genes (DEGs) number of the most enriched gene ontology (GO) term. C, GO enrichment analysis showing significantly enriched cellular process‐related pathways. It showed the *x*‐axis is the −log (*P*‐value), the size of the points corresponds to the percentage of the number of significant DEGs and the number of all the sequenced genes involved in the particular pathway. The larger the point is, the more genes in that pathway are involved in the MFS pathological process. D, Heatmaps showing the transcriptional level of genes enriched in cellular senescence

To further verify the senescence of MFS‐VPCs, we performed genome‐wide RNA sequencing (RNA‐seq). A total of 1724 up‐regulated genes and 2555 down‐regulated genes were obtained in MFS‐VPCs relative to control‐VPCs (Figure 3A). Gene ontology term enrichment analysis showed that cellular process contains the highest number of significant differentially expressed genes (both up‐ and down‐regulated genes) (Figure 3B). Then, we checked the enrichment analysis on the cellular process‐related pathways and found that cell cycle, cellular senescence and signalling pathways regulating the pluripotency of stem cells were significantly enriched (Figure 3C). We further examined these pathways and established that 81 differentially expressed genes between control‐VPCs and MF‐VPCs belong to the cellular senescence pathway, one of the top‐ranked cellular process‐related pathways (*P*‐value = 3.84e‐5), accounting for more than 25% of the total detected cellular senescence‐related genes. After removing novel DEGs, the samples could be correctly clustered according to the remaining known genes enriched in the cellular senescence pathway (Figure 3D). These data suggested a critical role of cellular senescence in the MFS‐VPCs.

### 3.4 TGF‐β1 regulates cellular senescence of VPCs in MFS

**Figure 4:**
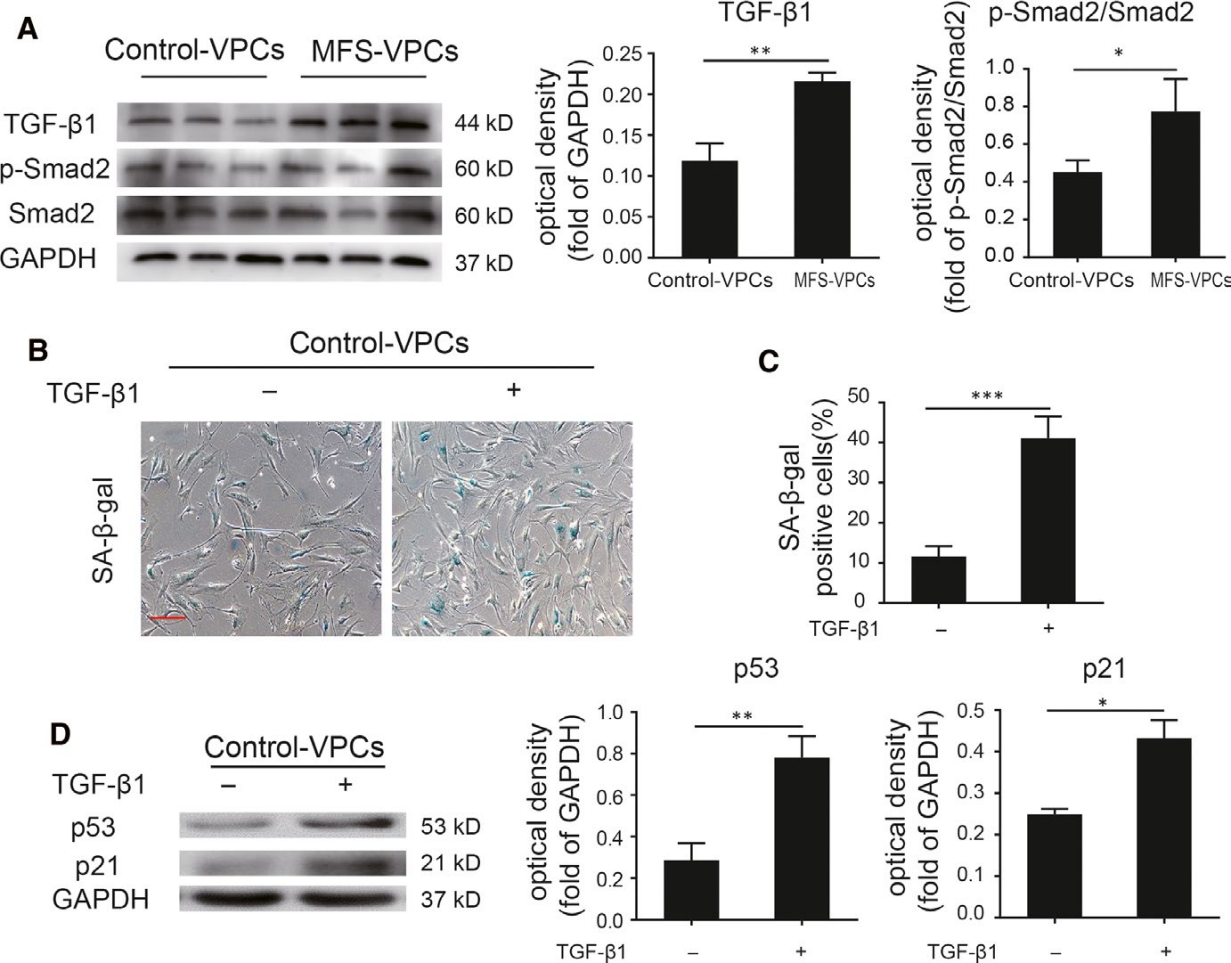
TGF‐β1 mediates cellular senescence of vascular progenitor cells (VPCs) in Marfan syndrome (MFS). A, Western blotting and quantitative analysis of the level of TGF‐β1 and p‐Smad2 protein in control‐VPCs and MFS‐VPCs. B, Representative images of SA‐β‐gal staining in control‐VPCs with or without TGF‐β1 treatment. C, The SA‐β‐gal positive cells in control‐VPCs with or without TGF‐β1 treatment were calculated and are presented as a percentage of total cells. D, Western blotting and quantitative analysis of the level of p53 and p21 protein in control‐VPCs with or without TGF‐β1 treatment. Data are expressed as mean ± SEM (n = 3). *P < 0.05, **P < 0.01, ***P < 0.001. Scale bar = 100 μm

Fibrillin‐1 defect can activate TGF‐β signalling and TGF‐β plays a critical role in mediating cell senescence. We examined the protein level of TGF‐β1 in control‐VPCs and MFS‐VPCs. Compared with control‐VPCs, the expression of TGF‐β1 was substantially increased in MFS‐VPCs (Figure 4A), indicating that TGF‐β1 may be involved in regulation of their cellular senescence. To support the effect of TGF‐β1 signalling in MFS‐VPCs, we measured the level of Smad2 phosphorylation. The results showed that the protein level of p‐Smad2 was greatly enhanced in MFS‐VPCs compared with control‐VPCs, suggesting enhanced TGF‐β1 activity in MFS‐VPCs (Figure 4A). To examine whether TGF‐β1 regulates the senescence of VPCs, we treated the control‐VPCs with TGF‐β1 for 48 hours. The results demonstrated that TGF‐β1 induced VPC senescence in a dose‐dependent manner, with administration of 50 ng/mL TGF‐β1 inducing the highest level (Figure S3). Based on the above results, we chose 50 ng/mL TGF‐β1 for further studies. Administration of TGF‐β1 not only enhanced SA‐β‐gal activity in control‐VPCs (Figure 4B,C), but also up‐regulated the protein level of p53 and p21 (Figure 4D). To further validate the role of TGF‐β1 in regulation of VPC senescence, we treated the MFS‐VPCs with TGF‐β1‐siRNA. Administration of TGF‐β1‐siRNA led to an obvious reduction in TGF‐β1, p53 and p21 protein level (Figure S4A) and SA‐β‐gal activity in MFS‐VPCs (Figure S4B,C). These data suggest that TGF‐β1 regulates the cellular senescence of VPCs.

### 3.5 TGF‐β1 regulates VPC senescence via mitochondrial ROS generation

**Figure 5:**
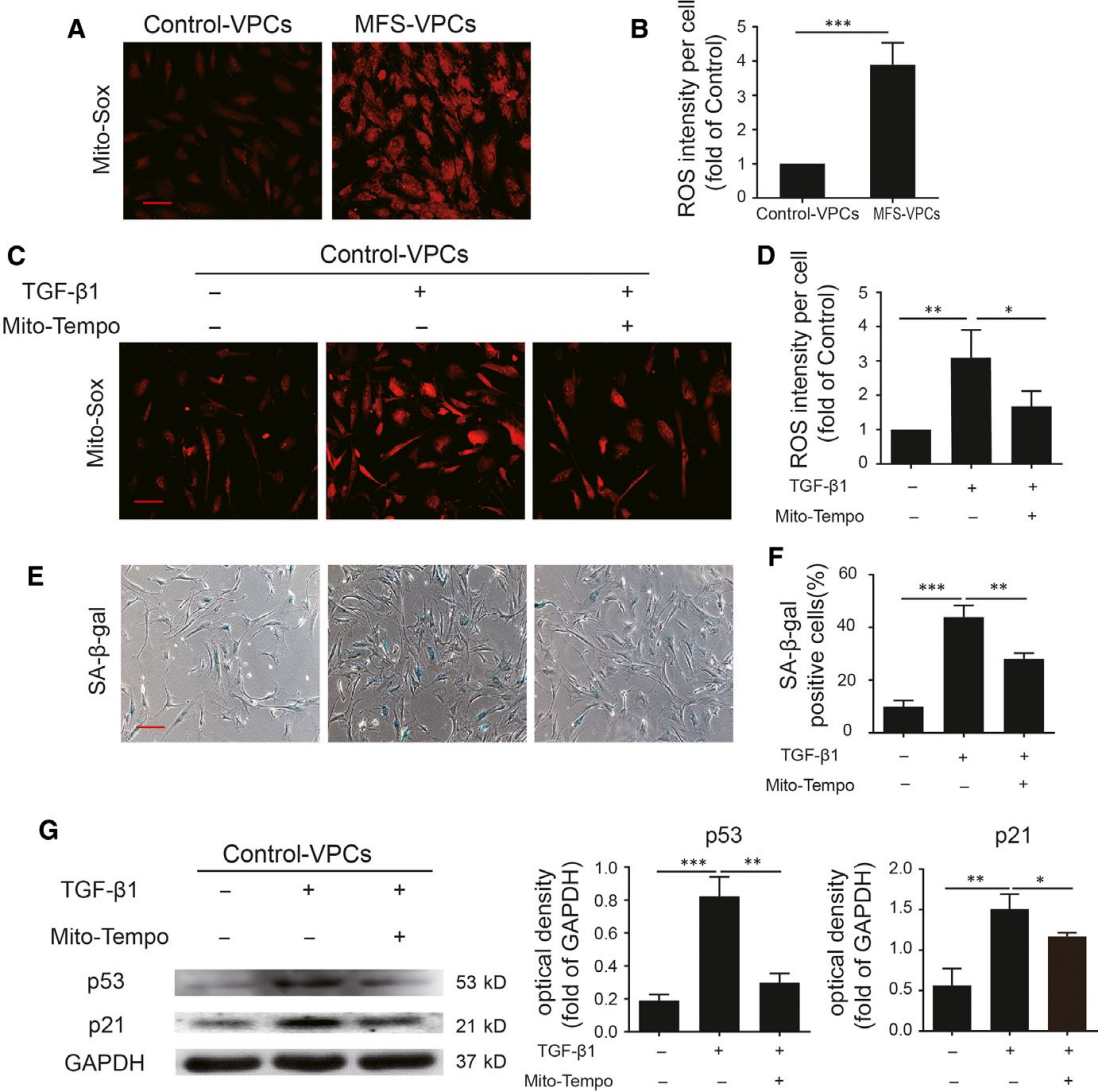
TGF‐β1 induces vascular progenitor cell (VPC) senescence via regulating mitochondrial reactive oxidative species (ROS) generation. A, Representative images of Mito‐Sox staining in control‐VPCs and Marfan syndrome (MFS)‐VPCs. B, Quantitative analysis of ROS generation in control‐VPCs and MFS‐VPCs. C, Representative images of Mito‐Sox staining in control‐VPCs that received TGF‐β1 or TGF‐β1 + Mito‐Tempo treatment. D, Quantitative analysis of ROS generation in control‐VPCs that received TGF‐β1 or TGF‐β1 + Mito‐Tempo treatment. E, Representative images of SA‐β‐gal staining in control‐VPCs that underwent TGF‐β1 or TGF‐β1 + Mito‐Tempo treatment. F, The SA‐β‐gal positive cells in control‐VPCs that received TGF‐β1 or TGF‐β1 + Mito‐Tempo treatment were calculated and are presented as a percentage of total cells. G, Western blotting and quantitative analysis of the level of p53 and p21 protein in control‐VPCs that received TGF‐β1 or TGF‐β1 + Mito‐Tempo treatment. Data are expressed as mean ± SEM (n = 3). *P < 0.05, **P < 0.01, ***P < 0.001. Scale bar = 100 μm

Accumulating evidence shows that elevation of reactive oxidative stress (ROS) generation contributes to cellular senescence.^27^, ^28^ We first tested the mitochondrial ROS generation in control‐VPCs and MFS‐VPCs by Mito‐sox staining (Figure 5A). Compared with control‐VPCs, ROS generation was significantly up‐regulated in MFS‐VPCs, indicating that ROS generation may participate in the regulation of VPC senescence (Figure 5B). Subsequently, we used the Mito‐Tempo, a mitochondria‐targeted antioxidant, to treat the MFS‐VPCs for 48 hours. Administration of Mito‐Tempo not only led to decreased ROS generation (Figure S5A,B), but also attenuated cellular senescence as demonstrated by reduced SA‐β‐gal activity in MFS‐VPCs (Figure S5C,D). Furthermore, Mito‐Tempo treatment down‐regulated the protein level of p53 and p21 in MFS‐VPCs (Figure S5E). To further verify whether TGF‐β1 induces VPC senescence via ROS generation, we examined the ROS generation in TGF‐β1‐treated control‐VPCs. The level of ROS (Figure 5C,D) and SA‐β‐gal activity was dramatically increased in TGF‐β1‐treated control‐VPCs (Figure 5E,F). Mito‐Tempo inhibited the increase of ROS (Figure 5C,D) and reversed the SA‐β‐gal activity induced by TGF‐β1 in control‐VPCs (Figure 5E,F). Importantly, Mito‐Tempo treatment also down‐regulated the increase in p53 and p21 induced by TGF‐β1 in control‐VPCs (Figure 5G). These results suggest that TGF‐β1 regulates VPC senescence via ROS generation.

### 3.6 TGF‐β1 induces mitochondrial fusion in VPCs

**Figure 6:**
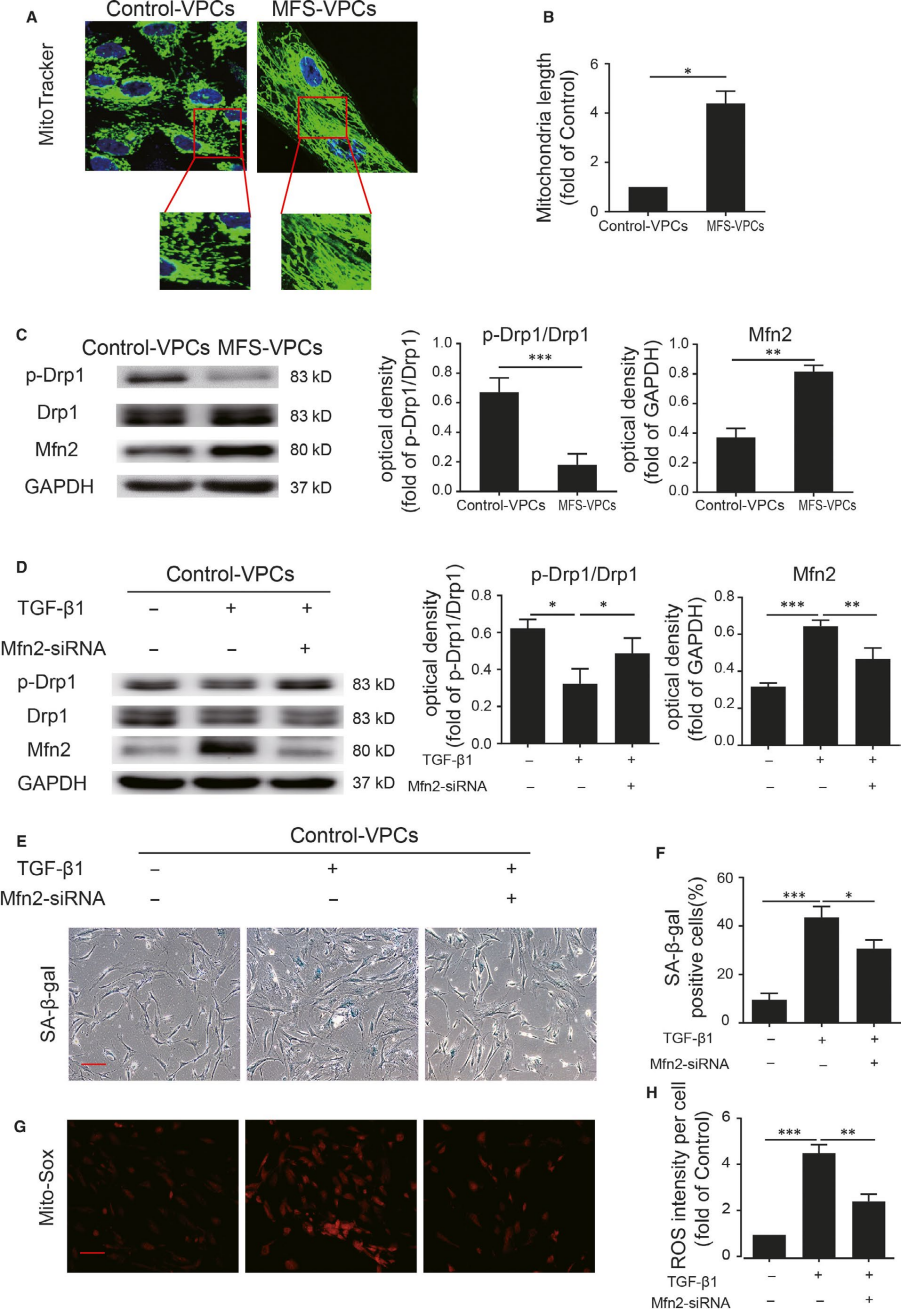
TGF‐β1 induces mitochondrial fusion in vascular progenitor cells (VPCs). A, Representative images of mitochondrial morphology determined by MitoTracker staining in control‐VPCs and Marfan syndrome (MFS)‐VPCs. B, The mitochondrial length in control‐VPCs and MFS‐VPCs was analysed. C, Western blotting and quantitative analysis of the level of p‐Drp1 and Mfn2 protein in control‐VPCs and MFS‐VPCs. D, Western blotting and quantitative analysis of the level of p‐Drp1 and Mfn2 protein in control‐VPCs that received TGF‐β1 or TGF‐β1 + Mfn2‐siRNA treatment. E, Representative images of SA‐β‐gal staining in control‐VPCs that received TGF‐β1 or TGF‐β1 + Mfn2‐siRNA treatment. F, The SA‐β‐gal positive cells in control‐VPCs that received TGF‐β1 or TGF‐β1 + Mfn2‐siRNA treatment were calculated and are presented as a percentage of total cells. G, Representative images of Mito‐Sox staining in control‐VPCs that received TGF‐β1 or TGF‐β1 + Mfn2‐siRNA treatment. H, Quantitative analysis of ROS generation in control‐VPCs that received TGF‐β1 or TGF‐β1 + Mfn2‐siRNA treatment. Data are expressed as mean ± SEM (n = 3). *P < 0.05, **P < 0.01, ***P < 0.001. Scale bar = 100 μm

It has been reported that abnormal mitochondrial dynamics are closely associated with mitochondrial ROS generation.^29^ We examined the mitochondrial morphology in control‐VPCs and MFS‐VPCs using MitoTracker staining. Surprisingly, compared with control‐VPCs, MFS‐VPCs exhibited increased mitochondrial length (Figure 6A,B). To our knowledge, Drp1 leads to mitochondrial fission whereas Mfn2 is correlated with mitochondrial fusion. Therefore, we measured the expression level of p‐Drp1 and Mfn2 in control‐VPCs and MFS‐VPCs. Western blotting showed that compared with control‐VPCs, Mfn2 was greatly enhanced whereas p‐Drp1 was markedly reduced in MFS‐VPCs (Figure 6C), indicating mitochondrial fusion in MFS‐VPCs. Subsequently, we tested whether mitochondrial fusion affected mitochondrial ROS generation by treating MFS‐VPCs with Mfn2‐siRNA. Mfn2‐siRNA treatment also inhibited MFS‐VPC senescence (Figure S6A,B). Moreover, Mfn2‐siRNA administration significantly reduced mitochondrial ROS (Figure S6C,D). Western blotting showed that Mfn2‐siRNA administration lead to an increase in p‐Drp1 level and a decrease in Mfn2 level in MFS‐VPCs (Figure S6E). Nevertheless, it was not clear whether TGF‐β1 induced ROS generation via regulation of mitochondrial dynamics. TGF‐β1 treatment significantly increased mitochondrial fusion as demonstrated by increased Mfn2 and decreased p‐Drp1 in control‐VPCs whereas Mfn2‐siRNA administration abrogated this effect (Figure 6D). Importantly, Mfn2‐siRNA administration also reduced cellular senescence (Figure 6E,F) and TGF‐β1‐induced ROS generation (Figure 6G,H). Collectively, these data show that TGF‐β1 induces mitochondrial ROS generation via regulation of mitochondrial dynamics.

### 3.7 AMPK signalling is involved in TGF‐β1 mediation of mitochondrial dynamics

**Figure 7:**
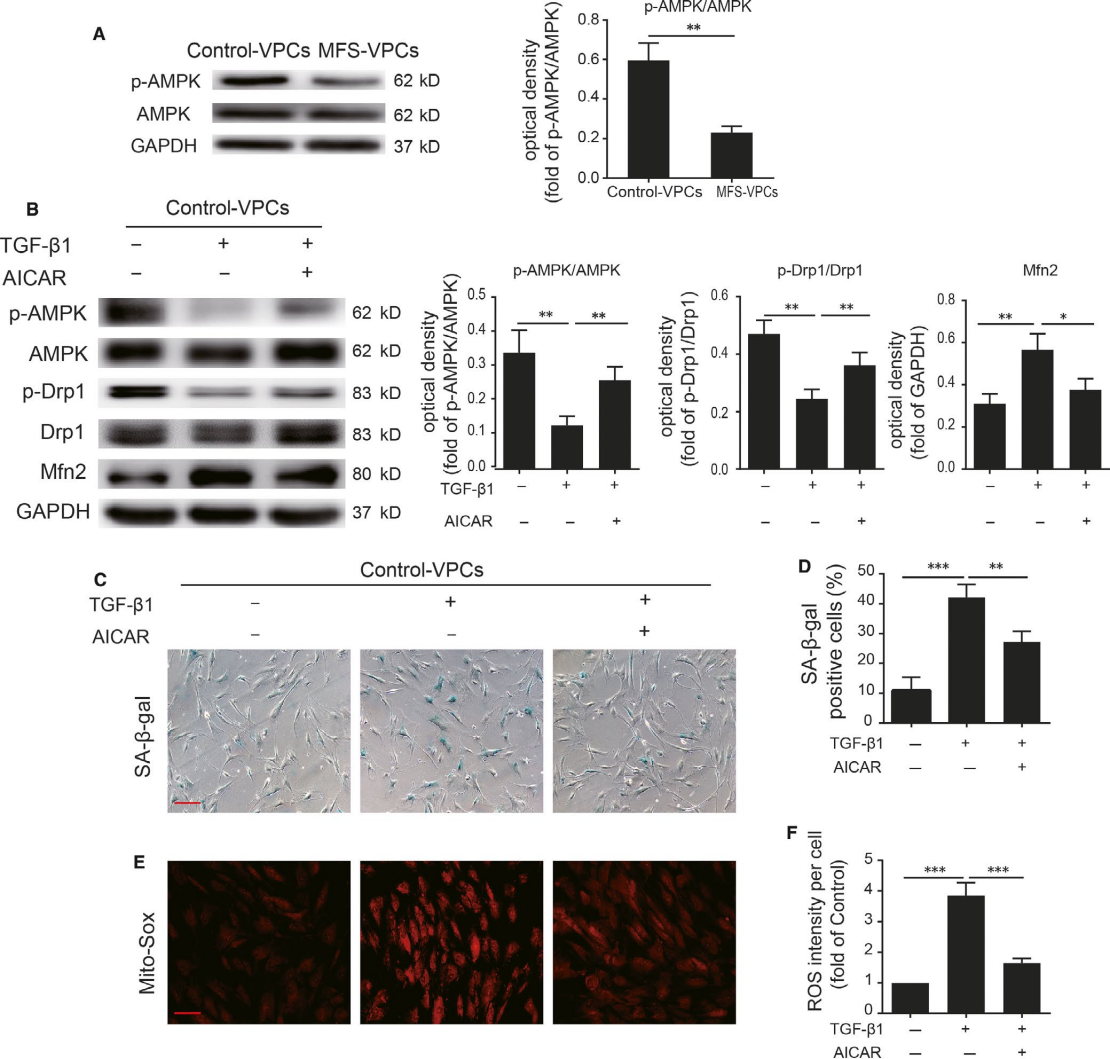
Adenosine monophosphate‐activated protein kinase (AMPK) signalling is involved in TGF‐β1 mediation of mitochondrial dynamics in vascular progenitor cells. A, Western blotting and quantitative analysis of the level of p‐AMPK protein in control‐vascular progenitor cells (VPCs) and Marfan syndrome (MFS)‐VPCs. B, Western blotting and quantitative analysis of the level of p‐AMPK, p‐Drp1 and Mfn2 protein in control‐VPCs that received TGF‐β1 or TGF‐β1 + AICAR treatment. C, Representative images of SA‐β‐gal staining in control‐VPCs that received TGF‐β1 or TGF‐β1 + AICAR treatment. D, The SA‐β‐gal positive cells in control‐VPCs that received TGF‐β1 or TGF‐β1 + AICAR treatment were calculated and are presented as a percentage of the total cells. E, Representative images of Mito‐Sox staining in control‐VPCs that received TGF‐β1 or TGF‐β1 + AICAR treatment. F, Quantitative analysis of ROS generation in control‐VPCs that received TGF‐β1 or TGF‐β1 + AICAR treatment. Data are expressed as mean ± SEM (n = 3). *P < 0.05, **P < 0.01, ***P < 0.001. Scale bar = 100 μm

The AMPK signalling pathway plays a critical role in regulation of mitochondrial dynamics.^30^ We therefore examined AMPK activation in control‐VPCs and MFS‐VPCs. We found that p‐AMPK was markedly reduced in MFS‐VPCs compared with control‐VPCs (Figure 7A). To further verify whether AMPK signalling participates in regulation of mitochondrial dynamics, we treated MFS‐VPCs with AICAR, an activator of AMPK. AICAR treatment significantly enhanced p‐AMPK and p‐Drp1 protein level and reduced Mfn2 protein level (Figure S7A) in MFS‐VPCs, indicating that AMPK signalling may be involved in the regulation of mitochondrial dynamics. Moreover, AICAR treatment attenuated cellular senescence (Figure S7B,C) and reduced mitochondrial ROS generation in MFS‐VPCs (Figure S7D,E). Subsequently, we tested whether TGF‐β1 mediated mitochondrial dynamics via AMPK signalling. Administration of TGF‐β1 significantly reduced AMPK activation, p‐Drp1 and increased Mfn2 expression whereas AICAR reversed these effects in control‐VPCs (Figure 7B). Furthermore, AICAR treatment attenuated TGF‐β1‐induced cellular senescence (Figure 7C,D) and ROS generation in control‐VPCs (Figure 7E,F).

## 4 DISCUSSION

Vascular stem cells/progenitor cells in the adventitia are essential for maintaining vessel integrity and function. The function of these cells isolated from a diseased donor declines although the underlying mechanisms remain unclear. In this study, we demonstrated that VPCs isolated from patients with MFS exhibited cellular senescence. We also found that TGF‐β1, via regulation of mitochondrial dynamics, induced VPC cellular senescence by elevation of mitochondrial ROS generation. More importantly, AMPK signalling was involved in TGF‐β1 mediation of mitochondrial dynamics.

Over the past decade, accumulating evidence has highlighted that a population of stem/progenitor cells exists in the sub‐endothelial zone and the adventitial zone of the vessel wall.^31^, ^32^, ^33^ These adventitial cells including the stem/progenitor cells play an essential role in maintaining physiological function of the vessel. These stem/progenitor cells express a panel of markers that define the progenitor cells such as Sca‐1, CD34, Flk‐1 or c‐kit.^18^ These cells usually are in a quiescent state in the vessel but can be activated by a pathophysiological condition to mediate tissue repair. In an injured vessel, these VPCs can migrate into the media or the intima to differentiate into SMCs or endothelial cells, participating in repair of the vessel to restore function.^18^, ^33^ In this study, we also found a population of VPCs expressing c‐kit in the adventitia of the aortic root. We revealed that as well as c‐kit, these cells expressed similar surface markers to mesenchymal stem cells such as CD90 and CD105. Importantly, these VPCs also can differentiate into adipocytes and osteocytes.

Cardiovascular complications are the major clinical feature of MFS. MFS patients usually suffer from aortic aneurysms that can result in rupture and consequent high mortality.^13^ Vascular injury can impair the function of VPCs resident in the adventitia, leading to reduced functional recovery of the vessel.^34^ Nevertheless, the function of VPCs in patients with MFS has not been examined. Here, we found that the number of VPCs in MFS patients was greatly decreased compared with that of control donors. Notably, these VPCs exhibited increased cell size and SA‐β‐gal activity along with a decreased migration and differentiation capacity, indicating cellular senescence. To further verify the cellular senescence of MFP‐VPCs, we performed RNA sequencing to analyse the transcriptomics. The results demonstrated that several cellular process‐related pathways including cell cycling and cellular senescence were significantly enriched in MFP‐VPCs compared with control‐VPCs, suggesting that MFP‐VPCs are senescent. Nevertheless, the potential mechanisms underlying cellular senescence of VPCs in MFS patients remain to be further elucidated.

It has been shown that TGF‐β plays a very important role in the pathogenesis of MFS.^35^, ^36^ FBN1 mutation leads to excessive activation of TGF‐β, resulting in activation of a cascade signalling pathway. TGF‐β1 activation can lead to ROS accumulation, contributing to fibroblast senescence; meanwhile excessive ROS can in turn further activate TGF‐β1, forming a positive TGF‐β1‐ROS autocrine loop.^37^ In this study, we showed that administration of TGF‐β1 could induce VPC cellular senescence via ROS generation and treatment of Mito‐Tempo attenuated this process, suggesting that TGF‐β1‐induced ROS accumulation is responsible for VPC senescence. Nevertheless, the relationship between TGF‐β1 and ROS generation has not been fully elucidated.

It is well known that mitochondria are the major source of ROS production. There is increasing recognition that altered mitochondrial dynamics are responsible for ROS overproduction. Depletion of protein disulfide isomerase A1 (PDIA1) promotes mitochondrial fission and elevates ROS generation, driving the senescence of endothelial cells.^38^ In contrast, abnormally elongated mitochondria induce ROS production, thereby triggering mammalian senescence.^39^ Furthermore, depletion of OPA1 leads to mitochondrial fission and rescues cells from senescence‐associated phenotypic changes.^39^ Senescent human adipose‐derived mesenchymal stem cells exhibit increased mitochondrial elongation and ROS generation, suggesting that mitochondrial fusion contributes to cellular senescence.^40^ Thus, mitochondrial fusion or fission can initiate cellular senescence and may be cell type‐ and context‐specific. However, the connection between mitochondrial fusion and ROS remains unclear. Increased ROS may lead to further mitochondrial fusion, causing further elevation in ROS generation, thus forming a vicious loop. In this study, we showed that compared with control‐VPCs, the mitochondria in MFS‐VPCs were greatly elongated. TGF‐β1 treatment increased Mfn2 expression and decreased p‐Drp1 in control‐VPCs. Nevertheless, Mfn2‐siRNA administration inhibited TGF‐β1‐induced mitochondrial fusion and ROS generation, and thus attenuated the cellular senescence of control‐VPCs. These results support the notion that TGF‐β1 induces VPC senescence via regulation of mitochondrial fusion.

The next question is how TGF‐β1 regulates mitochondrial fusion in VPCs. Previous studies have demonstrated that AMPK signalling mediates mitochondrial dynamics.^41^, ^42^ AMPK activation can phosphorylate MFF, a critical receptor protein for Drp1, to induce mitochondrial fission.^30^ We found that p‐AMPK was significantly reduced in MFS‐VPCs, combined with mitochondrial fusion in VPCs. We presumed that down‐regulation of p‐AMPK could induce mitochondrial dysfunction. It has been reported that AMPK activation induces mitochondrial dysfunction, leading to human fibroblasts senescence.^43^ In contrast, in this study, we found that treating MFS‐VPCs with AICAR reversed mitochondrial fusion and attenuated cellular senescence, suggesting that AMPK activation inhibits MFS‐VPCs senescence. This contradictory phenomenon may be cell type‐ and stimuli‐dependent. It has been demonstrated that TGF‐β1 activity impairs mitochondrial function in human skeletal muscle cells via suppression of AMPK activation.^44^ Furthermore, the activation of AMPK can inhibit TGF‐β1 release.^45^ Collectively, there seems to be a positive feedforward mechanism by which TGF‐β1 inhibits AMPK activation, that in turn down‐regulation of AMPK activation increases TGF‐β1 release. Consistent with this finding, in this study we showed that administration of TGF‐β1 significantly inhibited AMPK activation, leading to mitochondrial fusion. AICAR treatment abrogated TGF‐β1‐induced mitochondrial fusion and senescence in control‐VPCs.

There are also some limitations in this study. First, in addition to oxidative stress, telomere shortening or abnormal autophagy contributes to cellular senescence. Whether TGF‐β1 induces VPC senescence via regulation of telomere or autophagy requires further investigation. Second, whether targeting TGF‐β1 can enhance the therapeutic effects of VPCs isolated from MFS needs to be examined in a mouse model of MFS. Third, excessive production of proinflammatory cytokines is the major characteristic of vascular injury in MFS. Whether senescent VPCs contribute to inflammation in the aortic wall requires investigation. Last but not least, to support the role of FBN1 defect in VPCs senescence of MFS patients, whether reversion of fibrillin mutation can rescue the VPCs senescence needs to be examined.

Our results demonstrate that TGF‐β1 induced mitochondrial fusion, via suppression of AMPK signalling, leading to cellular senescence of VPCs from MFS patients. These findings pave the way to restoring the function of VPCs and provide a novel strategy for treatment of MFS.

## CONFLICT OF INTEREST

The authors declare no conflict of interest.

## AUTHOR CONTRIBUTIONS

HH performed the experiments, analysed the data and wrote the manuscript; YZ and XL: designed the experiments, analysed the data and wrote the manuscript; BY, GY, WY, YH: helped to perform the experiments and prepare the materials; ZL: helped to analyse the data; QL: provided the materials; XL: designed the study's analytic strategy. All authors read and approved the manuscript.
