# Supplementary material for: Vascular progenitor cell senescence in patients with Marfan syndrome
Source: J Cell Mol Med. 2019 Mar 28;23(6):4139–52. doi: 10.1111/jcmm.14301 (PMC6533473; doi:10.1111/jcmm.14301)
Supplement: Supplementary file 1 [file JCMM-23-4139-s001.docx]

**Supplemental Figure Legends:**

**Supplemental Figure 1: The senescence of control-VPCs and MFS-VPCs at passage 0 and passage 4.**

The senescence of control-VPCs and MFS-VPCs was examined by SA-β-gal staining at passage 0 and passage 4, respectively. Data are expressed as mean±SEM (n=3). ********p<0.001*. ns, not significant.

**Supplemental Figure 2: DNA damage in control-VPCs and MFS-VPCs.**

(A) Representative images of γH2AX staining in control-VPCs and MFS-VPCs. (B) The γH2AX-positive cells in control-VPCs and MFS-VPCs were counted and are presented as the percentage of total cells. Data are expressed as mean±SEM (n=3). ********p<0.001*.

**Supplemental Figure 3. TGF-β1 induces VPCs senescence in a dose-dependent manner.** VPCs were treated with for 48 hours and the senescence of VPCs was examined by SA-β-gal staining. Data are expressed as mean±SEM (n=3). ******p<0.05*, ********p<0.001*. ns, not significant.

**Supplemental Figure4. Administration of TGF-β1-siRNA ameliorates MFS-VPCs senescence.**

(A) Western blotting and quantitative analysis of the level of TGF-β1, p53 and p21 protein in control-siRNA- or TGF-β1-siRNA-treated MFS-VPCs. (B) Representative images of SA-β-gal staining in control-siRNA- or TGF-β1-siRNA-treated MFS-VPCs. (C) The SA-β-gal positive cells in control-siRNA- or TGF-β1-siRNA-treated MFS-VPCs were counted and are presented as the percentage of total cells. Data are expressed as mean±SEM (n=3). ******p<0.05*, *******p<0.01*. Scale bar=100μm.

**Supplemental Figure 5. Inhibition of mitochondrial ROS reduces cellular senescence of MFS-VPCs**

(A) Representative images of Mito-sox staining in MFS-VPCs with or without MitoTempo treatment. (B) Quantitative analysis of ROS generation in MFS-VPCs with or without MitoTempo treatment. (C) Representative images of SA-β-gal staining in MFS-VPCs with or without MitoTempo treatment. (D) The SA-β-gal positive cells in MFS-VPCs with or without MitoTempo treatment were counted and are presented as percentage of total cells. (E) Western blotting and quantitative analysis of the level of p53 and p21 protein in MFS-VPCs with or without MitoTempo treatment. Data are expressed as mean±SEM (n=3). **p<0.05*, ****p<0.001*. Scale bar=100μm.

**Supplemental Figure 6. Treatment of Mfn2-siRNA inhibits cellular senescence of MFS-VPCs**

(A) Representative images of SA-β-gal staining in control-siRNA- or Mfn2-siRNA-treated MFS-VPCs. (B) The SA-β-gal positive cells in control-siRNA- or Mfn2-siRNA-treated MFS-VPCs were counted and are presented as the percentage of total cells. (C) Representative images of Mito-sox staining in control-siRNA- or Mfn2-siRNA-treated MFS-VPCs. (D) Quantitative analysis of ROS generation in control-siRNA- or Mfn2-siRNA-treated MFS-VPCs. (E) Western blotting and quantitative analysis of the level of p-Drp1 and Mfn2 protein in control-siRNA- or TGF-β1-siRNA-treated MFS-VPCs . Data are expressed as mean±SEM (n=3). ******p<0.05*, *******p<0.01*. Scale bar=100μm.

**Supplemental Figure7. Activation of AMPK signaling inhibits cellular senescence of MFS-VPCs**

(A) Western blotting and quantitative analysis of the level of p-AMPK, p-Drp1 and Mfn2 protein in MFS-VPCs with or without AICAR treatment. (B) Representative images of SA-β-gal staining in MFS-VPCs with or without AICAR treatment. (C) SA-β-gal positive cells in MFS-VPCs with or without AICAR treatment are presented as the percentage of total cells. (D) Representative images of Mito-sox staining in MFS-VPCs with or without AICAR treatment. (E) Quantitative analysis of ROS generation in MFS-VPCs with or without AICAR treatment. Data are expressed as mean±SEM (n=3). ******p<0.05*, *******p<0.01, ***p<0.001.* Scale bar=100μm.

**Supplemental Figure 1.**


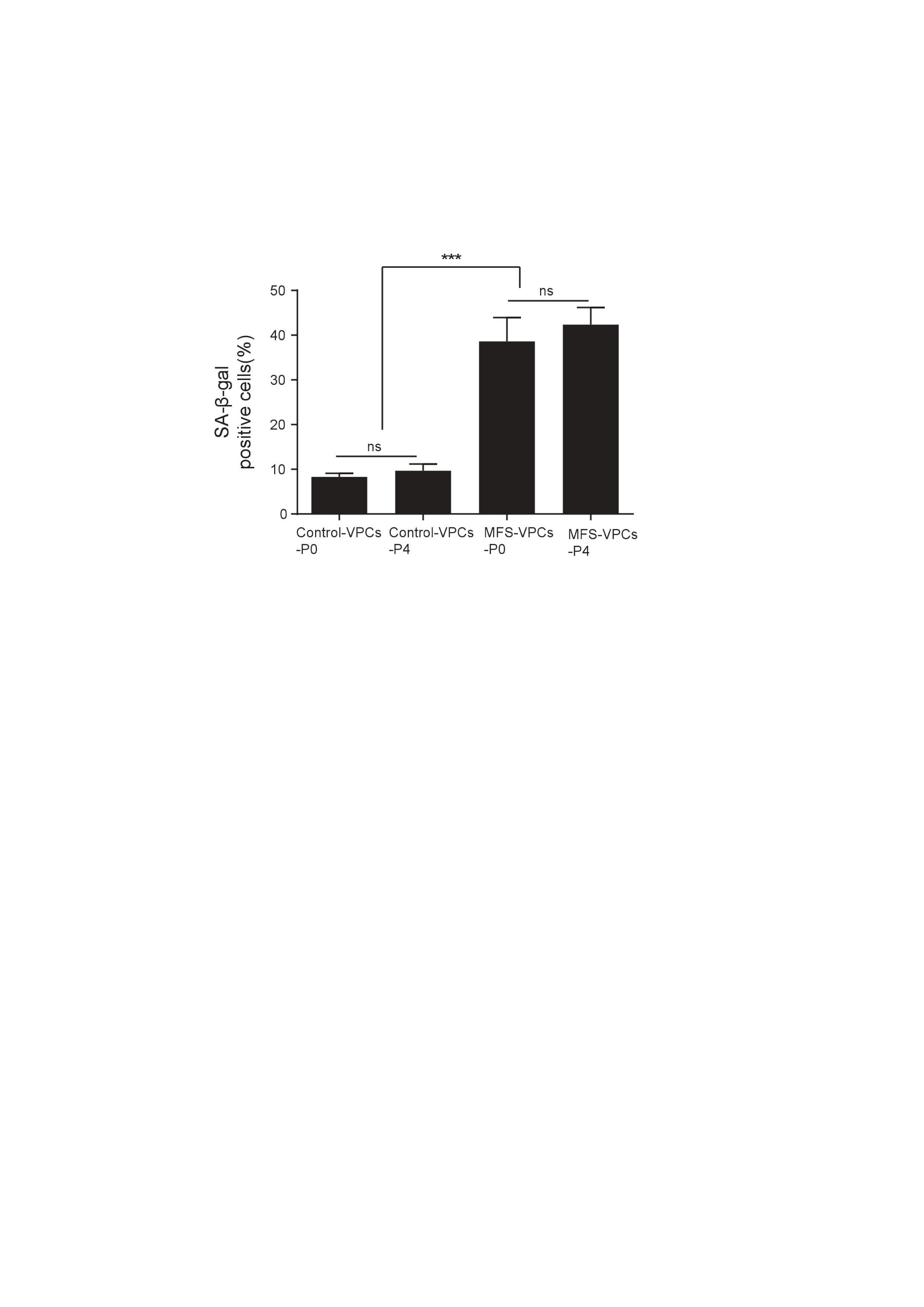


**Supplemental Figure 2**


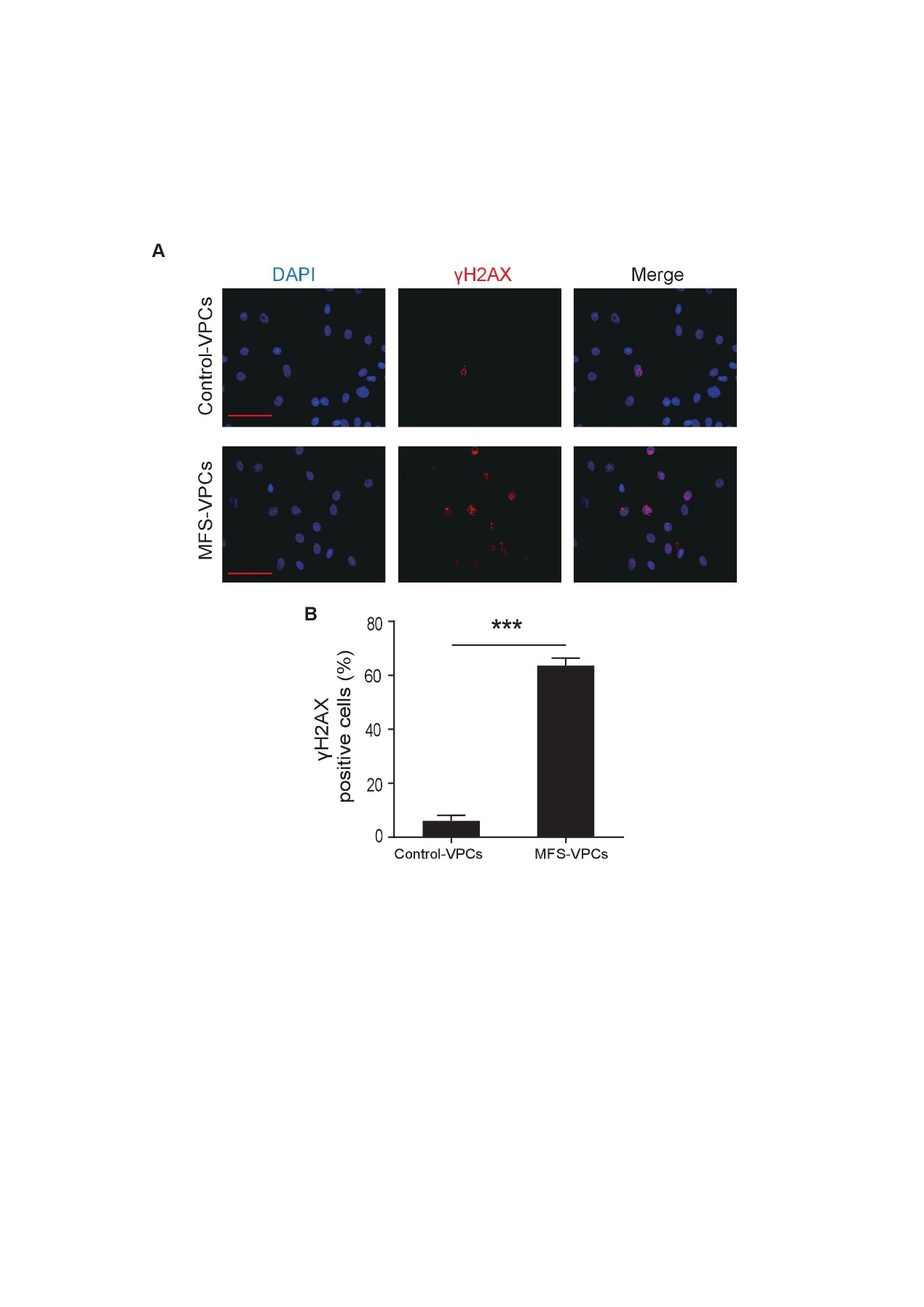


**Supplemental Figure 3**


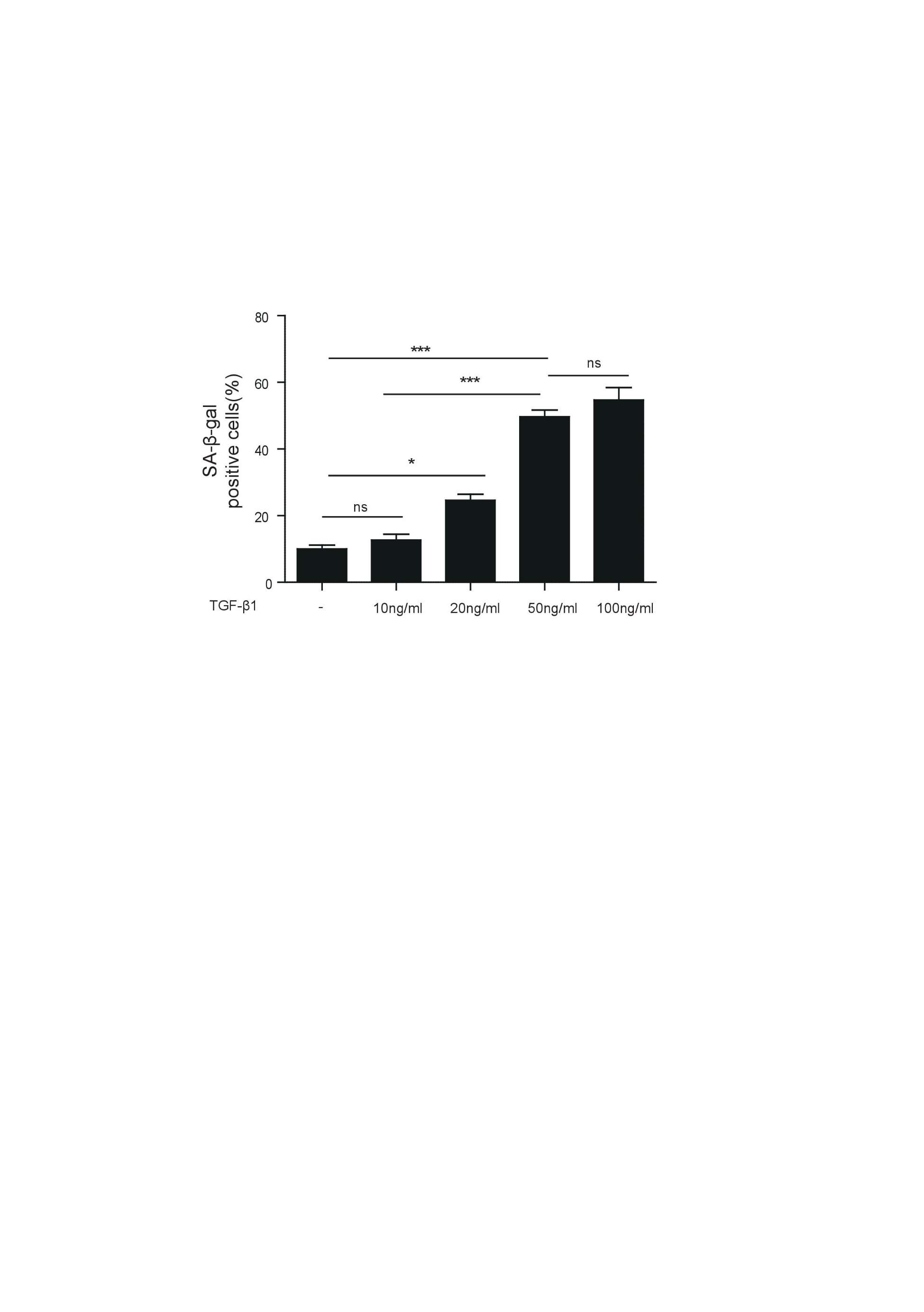


**Supplemental Figure 4**


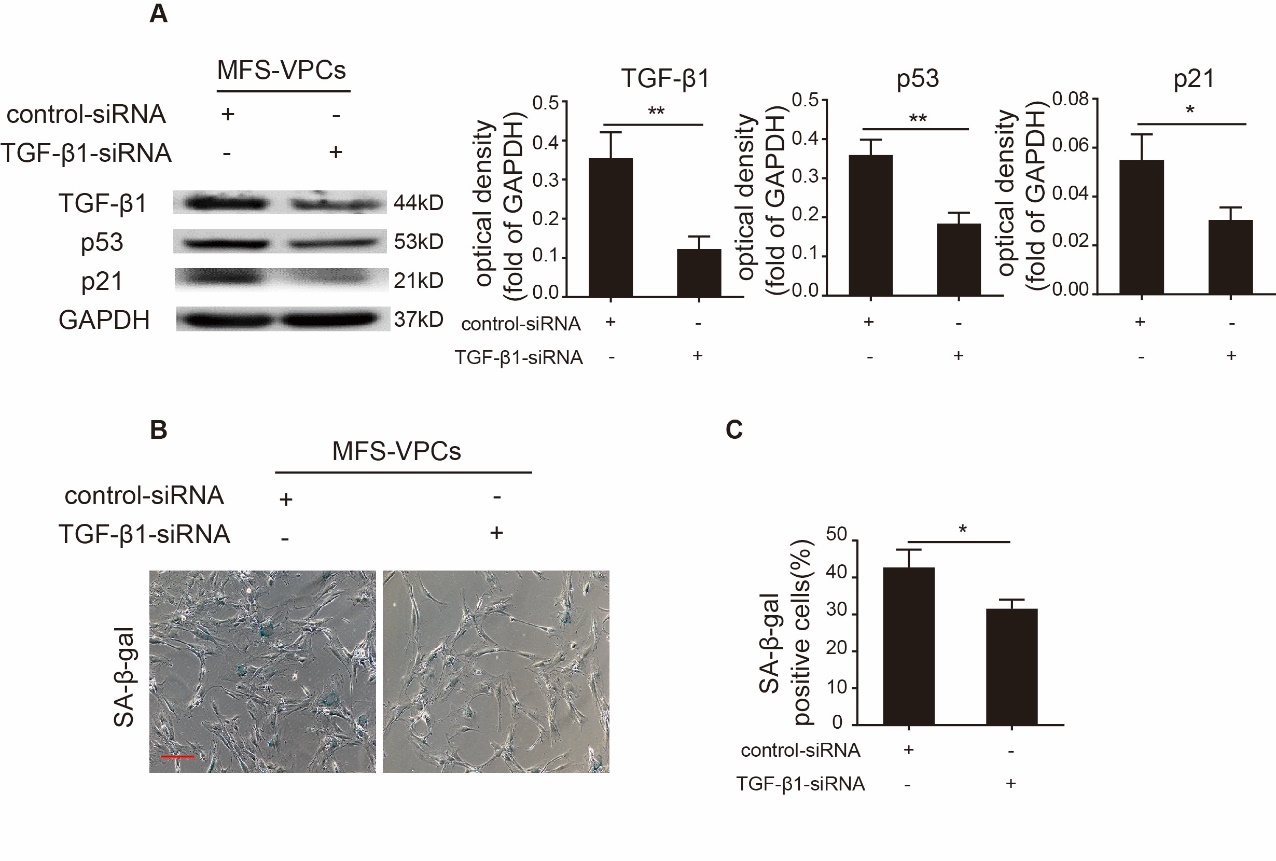


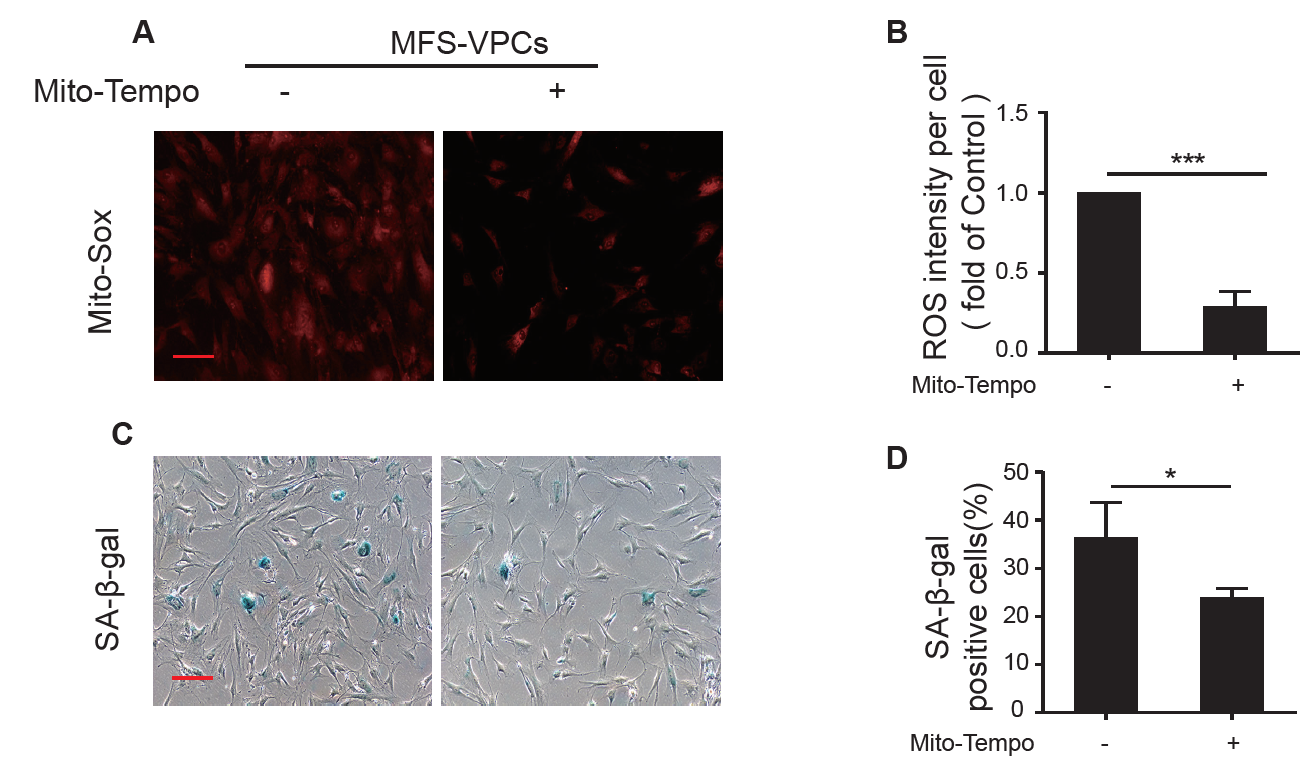
**Supplemental Figure 5**

**Supplemental Figure 6**


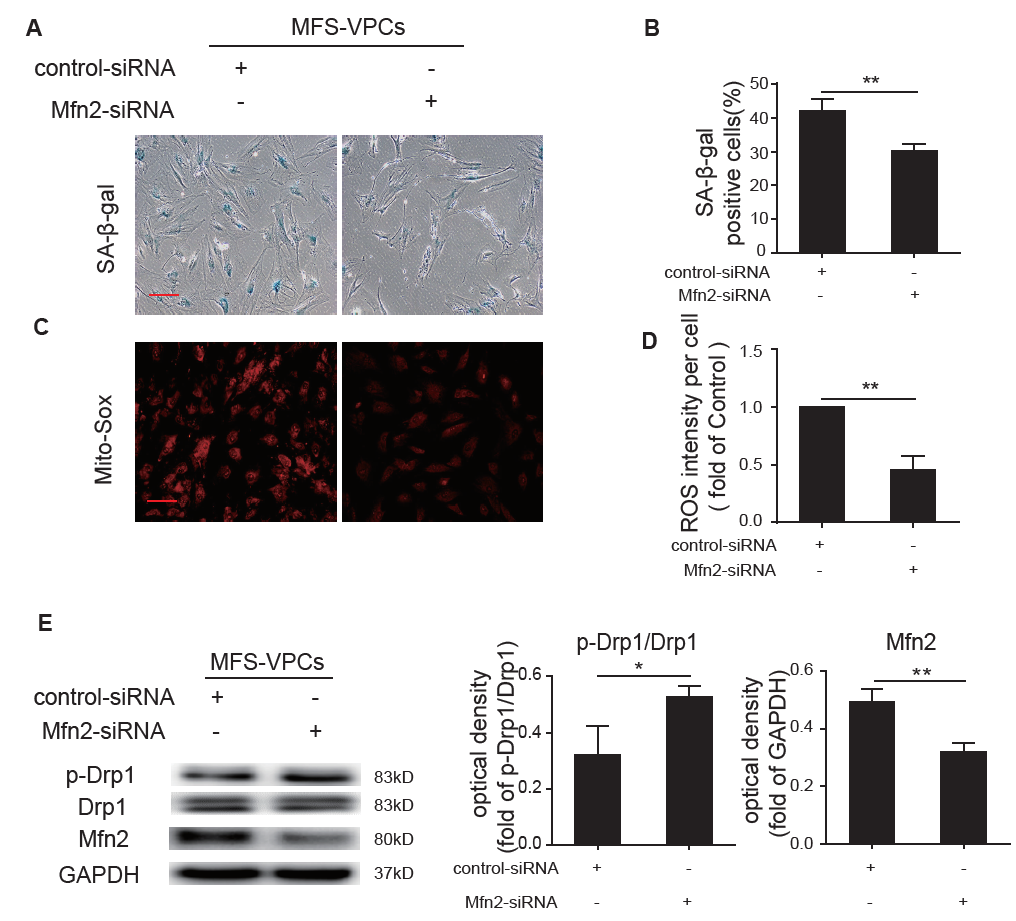


**
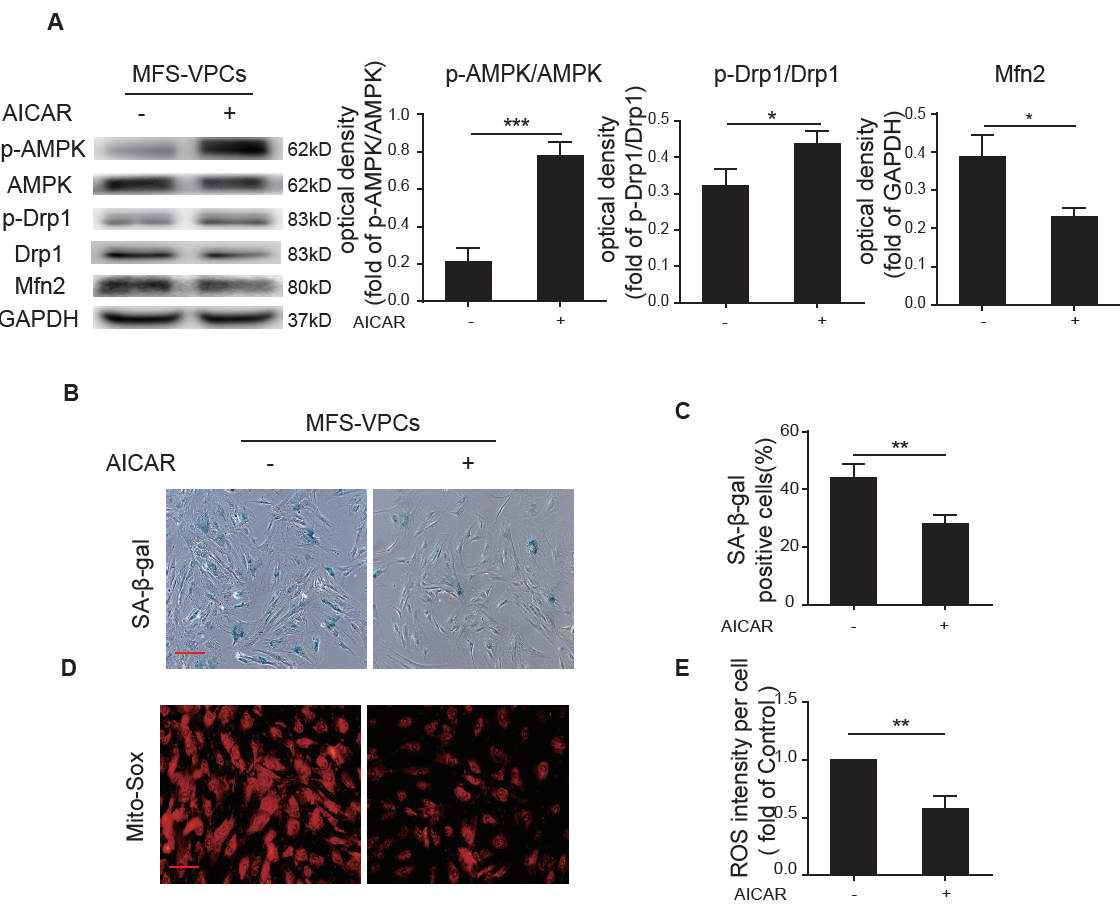
Supplemental Figure 7**
